# Genetic Diversity and Population Structure in Ethiopian Mustard (*Brassica carinata* A. Braun) as Revealed by Single Nucleotide Polymorphism Markers

**DOI:** 10.3390/genes14091757

**Published:** 2023-09-03

**Authors:** Misteru Tesfaye, Tileye Feyissa, Teklehaimanot Hailesilassie, Selvaraju Kanagarajan, Li-Hua Zhu

**Affiliations:** 1 Department of Plant Breeding, Swedish University of Agricultural Sciences, P.O. Box 190, 234 22 Lomma, Sweden; misteru.tesfaye.woldeyohannes@slu.sec (M.T.); selvaraju.kanagarajan@slu.se (S.K.); 2 Institute of Biotechnology, Addis Ababa University, Addis Ababa P.O. Box 1176, Ethiopia; tileye.feyissa@aau.edu.et (T.F.); teklehaimanot.hselassie@aau.edu.et (T.H.)

**Keywords:** *Brassica carinata*, genetic diversity, population structure, single nucleotide polymorphism marker

## Abstract

Ethiopian mustard (*Brassica carinata* A. Braun) is currently one of the potential oilseeds dedicated to the production for biofuel and other bio-industrial applications. The crop is assumed to be native to Ethiopia where a number of diversified *B. carinata* germplasms are found and conserved ex situ. However, there is very limited information on the genetic diversity and population structure of the species. This study aimed to investigate the genetic diversity and population structure of *B. carinata* genotypes of different origins using high-throughput single nucleotide polymorphism (SNP) markers. We used Brassica 90K Illumina Infinium^TM^ SNP array for genotyping 90 *B. carinata* genotypes, and a total of 11,499 informative SNP markers were used for investigating the population structure and genetic diversity. The structure analysis, principal coordinate analysis (PcoA) and neighbor-joining tree analysis clustered the 90 *B. carinata* genotypes into two distinct subpopulations (Pop1 and Pop2). The majority of accessions (65%) were clustered in Pop1, mainly obtained from Oromia and South West Ethiopian People (SWEP) regions. Pop2 constituted dominantly of breeding lines and varieties, implying target selection contributed to the formation of distinct populations. Analysis of molecular variance (AMOVA) revealed a higher genetic variation (93%) within populations than between populations (7%), with low genetic differentiation (PhiPT = 0.07) and poor correlation between genetic and geographical distance (R = 0.02). This implies the presence of gene flow (Nm > 1) and weak geographical structure of accessions. Genetic diversity indices showed the presence of moderate genetic diversity in *B. carinata* populations with an average genetic diversity value (*H_E_* = 0.31) and polymorphism information content (PIC = 0.26). The findings of this study provide important and relevant information for future breeding and conservation efforts of *B. carinata*.

## 1. Introduction

Ethiopian mustard (*B. carinata.* A. Braun) (carinata) is an allotetraploid (BBCC, 2*n* = 34) oilseed crop that has evolved through spontaneous interspecific hybridization between the diploid species *Brassica nigra* (BB, 2*n* = 16) and *Brassica oleracea* (CC, 2*n* = 18) [1,2]. It is believed to have originated in the highlands of Ethiopia, the adjoining portion of East Africa and the Mediterranean coast [3]. Cultivation of carinata as an oilseed and vegetable crop in Ethiopia and its neighboring countries dates back to 4–5 millennia ago [4]. Current evidence has shown that carinata has already been introduced to other continents, Europe, the Americas, Asia and Australia, due to its vast potential applications for biofuel and oleochemical industries [5,6].

In Ethiopia, carinata is cultivated mainly for its seed oil, either for cooking or during the preparation of traditional spicy foods [4]. The use of carinata oil for edible purposes is however limited due to its high erucic acid levels, ranging from 31 to 46%, which is much higher than the level recommended for edible oil (<2% in EU) [7]. Consequently, the development of carinata cultivars with high erucic acid has become an important research focus in recent years due to the rising demands of the crop for various industrial applications. Some of the industrial applications of the crop include biofuel and feedstock for oleo-chemical industries, as slip agents for plastic film production, lubricants and detergents [6,8,9,10,11]. 

Carinata has a high potential for use as low-carbon feedstock and in the production of jet biofuel for the aviation industry due to its attractive fatty acid profile, such as high erucic acid (>40%), low content of saturated fatty acids and minimal processing during refining [5,12]. This feature makes it suitable for sustainable aviation fuel production, thereby reducing greenhouse gas (GHG) emissions. The crop is thus receiving attention in the aviation industry that is responsible for 13% of GHG emissions from 45% of the total transport emission [13]. The use of carinata as drop-in renewable fuel has resulted in the expansion of the crop in the world due to the current strong demand to replace fossil fuel by biofuel [14]. The presence of good agronomic traits such as large seed size, drought and pod shatter resistance, and resistance to diseases, such as blackleg and *Alternaria* leaf spot, makes the crop more suitable in areas where other related crops fail to grow [15,16].

The multi-purpose nature of carinata has attracted breeders’ interest in mining genes responsible for important traits from the available gene pool of the crop. In Ethiopia, there are a number of carinata germplasms maintained in the gene bank of the Ethiopian Biodiversity Institute (EBI) and at research institutes or universities. Most of the germplasms at EBI are landraces or local cultivars, usually called accessions, while advanced breeding lines and released varieties are usually found at national or regional research centers and universities. These germplasms of carinata are important sources of genetic diversity and desirable genes for carinata trait improvement. Prior to the availability of DNA marker technologies, morphological and biochemical markers, including isozymes, were used to assess genetic diversity in crops [4,17]. Although such markers have provided useful genetic information for further breeding, they are limited in number and are highly influenced by environmental factors or the developmental stages of the species of concern [18]. Different DNA-based molecular marker techniques have been established and developed since 1980s, and some of them are currently used preferably for assessing genetic diversity due to their abundance in number and are not influenced by environmental conditions or developmental stages [19].

Compared with other major crops including oilseeds, there is relatively limited information available on genetic diversity and population structure for carinata using DNA-based molecular markers especially using high-throughput markers like single nucleotide polymorphisms (SNPs) [20]. In earlier studies, substantial genetic diversity was found in carinata using amplified fragment length polymorphism (AFLP) [21] and random amplified polymorphic DNA (RAPD) [22]. A low level of genetic diversity was obtained from 66 carinata accessions using AFLP markers [23] and from 78 carinata accessions using simple sequence repeat (SSR) markers [24]. A recent study by Khedikar et al. [25] has similarly revealed a low level of genetic diversity among 620 accessions using SNPs generated from genotyping by sequencing (GBS).

For SNP analysis, a genotype array of Brassica 90K Illumina Infinium SNP array, redesigned from a 60K array including 30,000 additional *Brassica* species B genome information, has been developed for genetic analysis of allotetraploid *Brassica* species [26,27]. This genotyping platform was employed at large to *B. napus* [28,29,30,31] and to some extent to its ancestral diploid *B. oleracea* [32,33] for genetic diversity analysis and genome-wide association mapping. However, the application of high-throughput genotyping in carinata is very limited, and so far, high-density SNP array has not been applied for genetic diversity studies of this crop to our best knowledge. This study aimed to assess the genetic diversity and population structure of selected carinata genotypes using high-density SNP markers.

## 2. Materials and Methods

### 2.1. Plant Material

**Figure 1:**
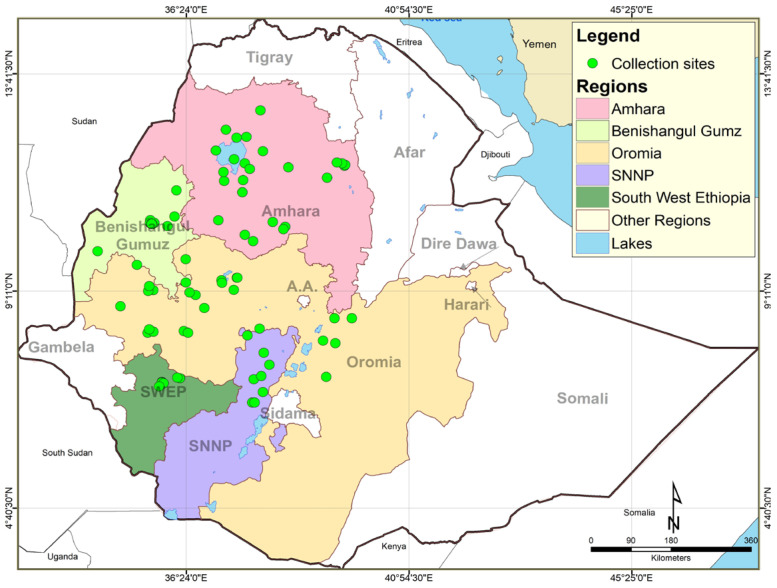
Geographical location of carinata accessions used in the study (SNNP refers to South Nation Nationalities Peoples Regional State).

A total of 90 carinata genotypes, including three sets of breeding materials, were used in this study (Table S1). The first set was composed of 75 accessions obtained from the Ethiopian Biodiversity Institute (EBI), which were collected from five major Regional States of Ethiopia, namely, Oromia, Amhara, South Nation and Nationalities and People (SNNPs), South West Ethiopian People (SWEP) and Benishangul-Gumuz (BNG). The geographical locations from where accessions were collected are shown in Figure 1. The second and third sets consisted of 10 and 5 advanced breeding lines and released varieties, respectively, which were acquired from the oilseeds breeding program of Holetta Agricultural Research Center (HARC).

### 2.2. Genotyping and SNP Marker Filtration

The 90 carinata genotypes were grown in biotron at 16 h photoperiod, 250 µmol/m^2^/s light intensity, 21/18 °C temperature (day/light) and 65% humidity at Swedish University of Agricultural Sciences (SLU). Genomic DNA of 4–5-week-old plants was extracted using the standard CTAB DNA extraction protocol as described by Clarke [34] with minor modifications. The quality of genomic DNA was checked using 1% agarose gel electrophoresis, and DNA concentration was measured fluorometrically using a Quantflour-ST (Promega GmbH, Walldorf, Germany). Genotyping was performed with the 90k Brassica array according to the manufacturer’s instructions by SGS Institute Fresenius GmbH Trait Genetics Section. The array was scanned with an iScan™ system (Illumina, Berlin, Germany), followed by data analysis with GenomeStudio 2.0 (Illumina), from which 77,970 SNP markers were extracted for further filtering.

Markers’ filtration and data imputation were conducted to get informative SNP markers. After removing a total of 23,586 monomorphic markers, SNP positions along with chromosomes were obtained from the reference sequence of *Brassica* species to construct a hapmap file for further analysis [35,36]. A total of 39,099 SNPs with >10% missing data and 3786 SNPs with <5% minor allele frequencies were excluded from further analysis using TASSEL software version 5.0 [37]. Finally, 11,499 informative markers were maintained for this study.

### 2.3. Population Structure

Population structure analysis was performed based on the filtered 11,499 SNP markers using STRUCTURE software version 2.3.4, which employs the Bayesian model clustering approach [38]. The number of subpopulations (K) was set from 1 to 10, and for each K, five runs were performed using a burn-in period of 50,000 iterations and 100,000 Markov Chain Monte Carlo (MCMC) repetitions. The best number of population (K-value) was identified based on ad hoc statistics (∆K), and the peak of ∆K value distribution was analyzed using web-based Structure Harvester software v0.694 [39]. Genotypes were classified into subpopulations based on their membership coefficients estimated using the STRUCTURE software version 2.3.4. We used the probability of membership coefficient >0.70 as threshold to assign genotypes to a specific subpopulation, and those genotypes with <0.70 membership coefficient were considered as admixture. Principal component analysis (PCA) was conducted by covariance standard approach using GenAlEX software version 6.5 [40]. We constructed a phylogenic tree by applying an unweighted pair group method with arithmetic mean (UPGMA) clustering based on Nei’s genetic distance [41] using PowerMarker software version 3.0 [42].

### 2.4. Genetic Diversity

Genetic diversity indices including genetic diversity (*D*) or expected heterozygosity (*H_E_*) and observed heterozygosity (*H_O_*) of SNP markers across subpopulations and sub-genomes were analyzed using PowerMarker software version 3.0 [42]. Analysis of molecular variance (AMOVA), genetic differentiation (PhiPT) and gene flow (Nm) were performed based on the number of distinct subpopulations (Pop1 and Pop2) obtained from the structure analysis using GenAlEX version 6.5 [40]. Additionally, Mantel test was conducted for the accessions collected from different agro-ecological regions using GPS data (latitude and longitude). For the Mantel test, we analyzed the genetic and geographical distance, followed by conducting the test using GenAlEX software version 6.5 [40].

### 2.5. Polymorphism Information Content (PIC)

Polymorphism information content (PIC) of 11,499 SNP markers was determined using PowerMarker software version 3.0 [42]. The PIC of the SNP markers was explained with its average value, based on the range set by Botstein et al. [43]. The SNP markers with a PIC value ≥ 0.5 are assumed to be highly informative, and those with values ranging 0.25–0.50 are moderately informative, while SNPs with PIC value less than 0.25 are slightly informative markers.

## 3. Results

### 3.1. SNP Analysis

**Figure 2:**
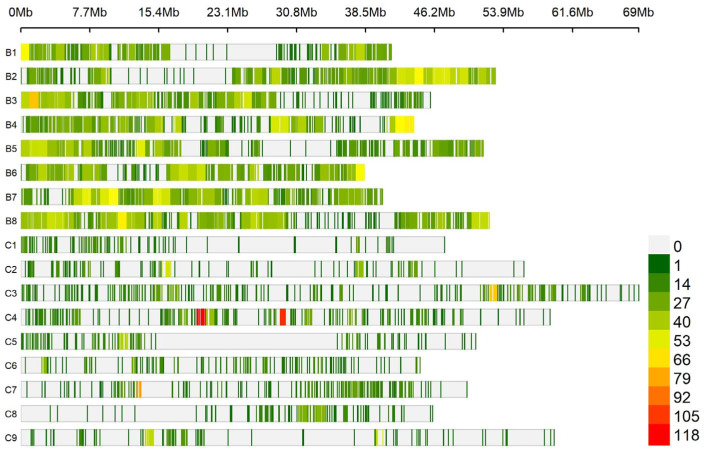
The density of 11,499 SNPs markers over 17 carinata chromosomes. The horizontal axis shows the chromosome length (MB); the different color depicts SNP density along with the number of SNPs within 1 MB window size.

**Figure 3:**
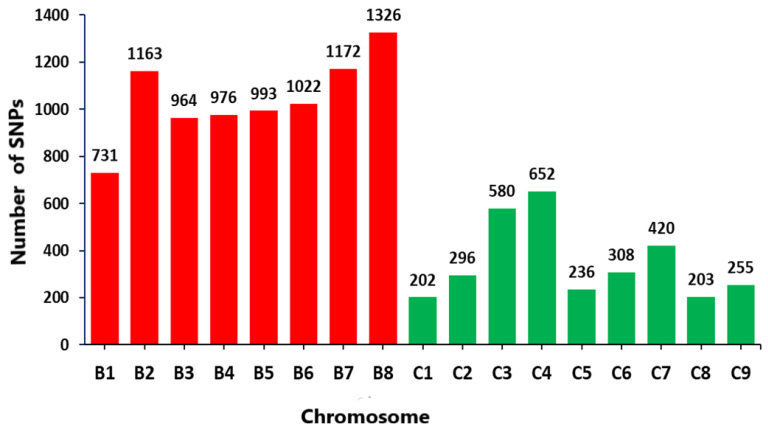
Distribution of 11,499 single nucleotide polymorphism (SNPs) across the B genome (red) and C genome (green) in the 90 carinata genotypes analyzed.

The SNP density was plotted using the R package CMplot (http://github.com/YinLiLin/R-CMplot, accessed on 3 April 2023). The SNP density plot indicated that 11,499 SNPs were distributed across 17 chromosomes, out of which 8 were from B genome and 9 were from C genome (Figure 2). The number of SNPs within 1 MB window size was large at C4 chromosome as described from the bottom right of Figure 2 with red color. This implies the presence of high density of SNPs in this chromosome region within 1 MB window size. Considering the whole genomic regions, the highest number of SNP markers were found at chromosome B8 (1326), while the least number of SNPs were observed at C1 (202) (Figure 3). Overall, 73% of the SNPs were located on the B genome and 27% SNPs were on the C genome.

The percentage of transition SNPs was found to be higher in both the B (52%) and C (19%) genomes as compared to transversion SNPs, which were 21% and 8.3% in B and C genomes, respectively (Table 1). Although the B genome showed only two types of transversion SNPs (i.e., A/C and G/T), their percentage was larger than the C genome, which contained all four types of transversion SNPs. In general, the SNP analysis indicated that the transition SNPs were more frequent than the transversion ones.

### 3.2. Population Structure

**Figure 4:**
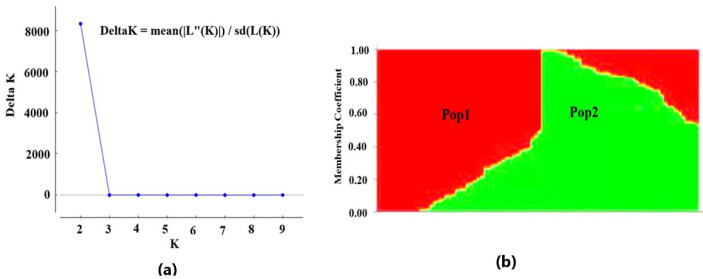
Population structure analysis of carinata genotypes. (**a**) Structure harvester Evanno’s test ∆K at K = 2. (**b**) Bar plot of structure analysis for K = 2 from STRUCTURE.

The 90 genotypes of carinata were grouped into two subpopulations based on the result of STRUCTURE analysis (∆K at K = 2) hereafter referred as Pop1 and Pop2 (Figure 4a,b). Pop1 constituted 49 genotypes, with 65% accessions sourced from five regions of Ethiopia. Among 90 genotypes, a larger percentage of accessions in Pop1 were from Oromia (84%), followed by SWEP (80%) and SNNP (60%) (Table 2). Almost half of the accessions from Amhara and Benishagul-Gumuz (BNG) were also included in Pop1. Unlike Pop1, Pop2 comprised 41 germplasms that included all advanced breeding lines (10) and released varieties (5) and the remaining 26 accessions. In terms of regional distribution versus proportion of accessions, Pop2 comprised 50% of the accession from BNG, 48% of accessions from Amhara, 40% from SNNP, 20% from SWEP and 16% from Oromia (Table 2). Bar plot of structure analysis at K = 3, K = 4 and K = 5 is presented as a supplement (Figure S1).

**Figure 5:**
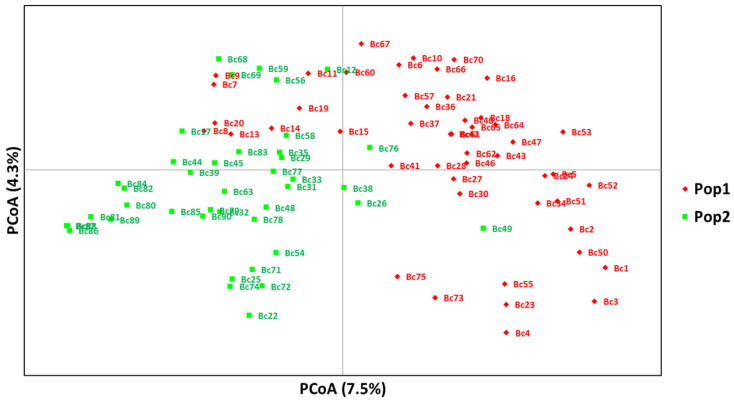
Principal coordinate analysis (PcoA) plot showing the two subpopulations (Pop1 and Pop2) clusters of the 90 carinata genotypes.

**Figure 6:**
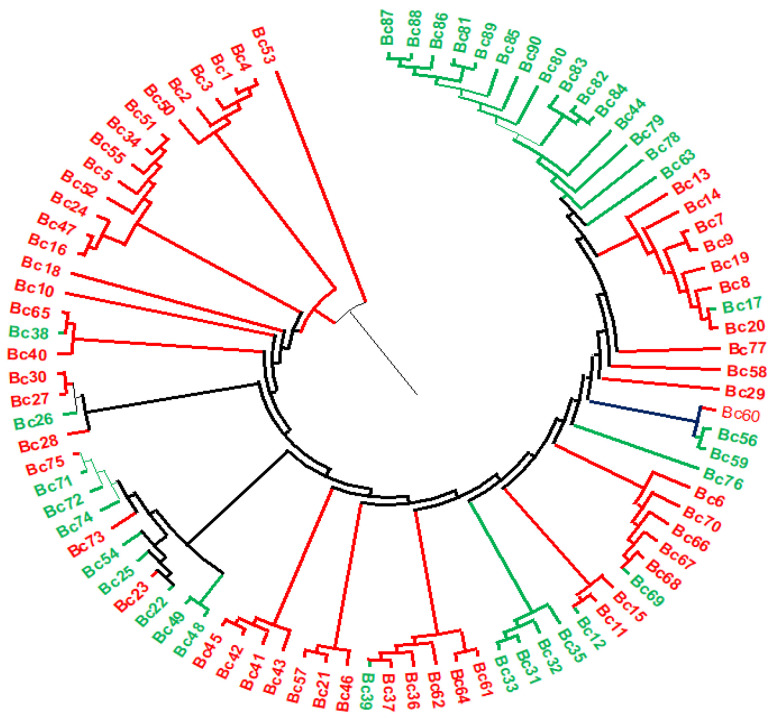
Phylogenic tree based on unweighted pair group method with arithmetic mean (UPGMA) that indicates the two subpopulations clusters Pop1 (red) and Pop2 (green) of the 90 carinata genotypes.

The principal coordinate analysis (PcoA) based on 11,499 SNPs clustered the 90 carinata populations into two subpopulations (Pop1 and Pop2) in similar fashion as that of the STRUCTURE analysis (Figure 5). The first two axes explained 12% (PCoA1 = 7.5 and PCoA2 = 4.3) of the total observed variation (Figure 5, Table S2). The phylogeny tree confirmed that the 90 carinata genotypes grouped into two district subpopulations (Pop1 and Pop2) (Figure 6).

### 3.3. Analysis of Molecular Variance

Analysis of molecular variance (AMOVA) was conducted based on the two distinct subpopulations determined from the STRUCTURE analysis. The analysis results revealed that variations within population accounted for 93% of the total variation, which was significantly higher than variations between populations (7%), as indicated in Table 3. The genetic differentiation between subpopulations (Pop1 and Pop2) was found to be poor (with PhiPT = 0.07) with significance at *p* < 0.001 and gene flow value (Nm) of 6.65 (Table 3).

### 3.4. Genetic Diversity

**Figure 7:**
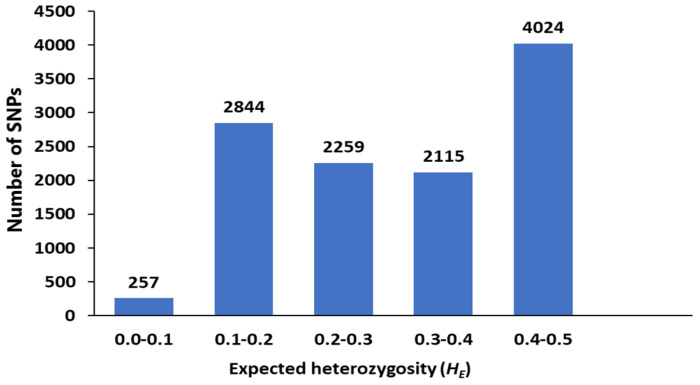
Distribution of genetic diversity (*H_E_*) of 11,499 single nucleotide polymorphism (SNP) markers in the 90 carinata genotypes.

A pattern of genetic diversity of carinata genotypes was revealed using genetic indices, gene diversity/expected heterozygosity (*H_E_*) and observed heterozygosity (*H_O_*) at individual genome (B or C), whole genome BC and subpopulations (Pop1 and Pop2) levels. The level of expected (*H_E_*) and observed (*H_O_*) heterozygosity in carinata was found to be 0.314 and 0.176, respectively, at the whole genome level (Table 4). There was no much difference based on the above-mentioned genetic indices between B genome (*H_E_* = 0.315, *H_O_* = 0.179) and C genome (*H_E_* = 0.309, *H_O_* = 0.168). In all cases, the observed heterozygosity was low compared to the expected heterozygosity. In the subpopulations, Pop1 showed almost the same level of *H_E_* but a higher level of *H_O_* than Pop2 (Table 3). In general, the results of genetic diversity indicate the presence of moderate diversity in the 90 carinata genotypes with an average value (*H_E_* = 0.314), and about 51% of SNPs showed *H_E_* above the average value (Figure 7, Table S3).

**Figure 8:**
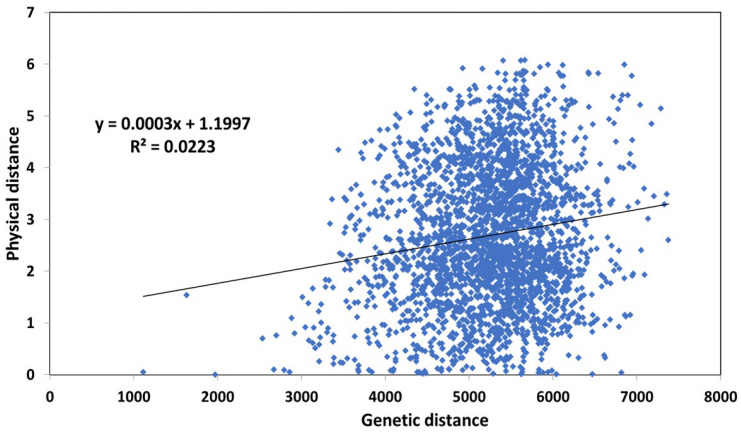
Mantel test for accessions based on the relationship between the genetic distance and geographical distance with reference to GPS data.

The Mantel test, which is used to demonstrate the spatial distribution of accessions, showed that the genetic and geographic matrices did not correlate as explained by low R-square value of 0.02 (Figure 8).

### 3.5. Polymorphism Information Content

**Figure 9:**
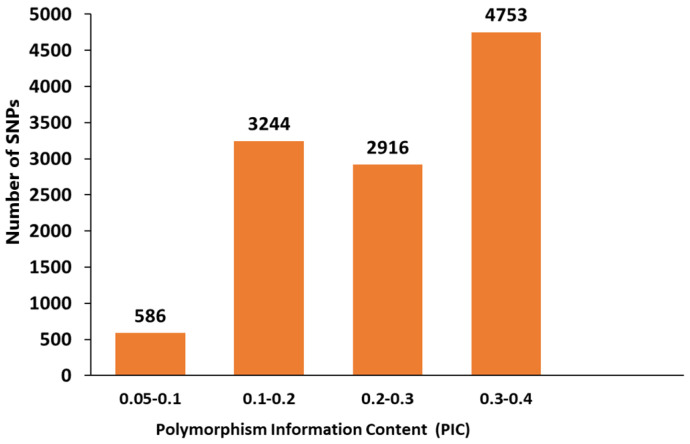
Distribution of single nucleotide polymorphism (SNP) markers with polymorphism information content (PIC) of the total 11,499 SNP markers of the 90 carinata genotypes.

Polymorphism information content (PIC) of 11,499 SNP markers was found within the range of 0.09 to 0.38 with an average value of 0.26 (Table S3). According to the range set by Botstein et al. [43], the SNP markers used in this study were found to be moderately informative. We identified 4753 SNP markers with PIC values within the range of 0.3–0.4, which are assumed to be more informative (Figure 9). PIC value of carinata markers across B and C genomes showed variability with the range of 0.18 to 0.30 with a mean value of 0.26 (Table 5). Although the number of SNPs was larger in the B genome (73%) than the C genome (27%), most of the chromosomes in the C genome had PIC values above the mean value. The larger PIC values were found at C5 (0.3) chromosome, followed by C7 (0.299) and C8 (0.292) chromosomes. Chromosomes B1 and B3 were found to have a small number of polymorphic SNPs of 731 and 964, respectively, showing PIC values above the mean.

## 4. Discussion

The hybridization arrays are believed to be powerful tools for generating high-quality markers that can be used for various applications in plant breeding. High-density SNP markers arrays were prominent in *B. napus* among *Brassica* species as they were first designed from genomic and transcriptome data of *B. napus* and its progenitors *B. oleracea* and *Brassica rapa* [26]. In this study, we employed Brassica 90K Illumina Infinium SNP arrays and obtained 77,970 SNP markers. After filtering, 11,499 high-quality markers were used to investigate the genetic diversity and population structure of the 90 carinata genotypes. The number of polymorphic SNPs in carinata was found to be larger in the B genome (8347) than in the C genome (3152). This finding is consistent with those of Zou et al. [44] and Khedikar et al. [25]. The presence of high polymorphic rate in the *Brassica* B genome could be related to the divergence of *B. nigra* genome (8 million years ago) prior to *B. oleracea* (4 million years ago) from their common ancestor, and thus the former species could have accumulated the higher level of mutations [45].

Another important finding from the SNP analysis was the frequency of substitution mutations that resulted in the two types of single nucleotide polymorphisms (SNPs), transition and transversion. High frequency of transition SNPs was found over transversion SNPs. This result agrees with the findings of closely related species of carinata such as *B. napus* [46,47,48], *Brassica juncea* [49], and *B. rapa* L. [50]. The causes of various polymorphism rates between the sub-genomes could be linked to selection pressure during domestication and breeding and/or new recombination or mutation rates [25,51,52].

As revealed from STRUCTURE analysis, the 90 carinata genotypes were grouped into two subpopulations (Pop1 and Pop2) whereby the majority of genotypes included in Pop1 were accessions collected from two regions, Oromia (84%) and South West Ethiopian People (SWEP) (80%). This might be due to the fact that those accessions obtained from the two regions share common features or might have been accessed from the nearby districts of the two regions. Interestingly, all the advanced breeding lines and released varieties were found in the other group, Pop2, along with 26 accessions, which are mainly from BNG, SNNP and Amhara regions (Table 2). The clustering of all breeding lines and varieties in Pop2 indicates the impact of targeted selection in the formation of distinct populations. The presence of 37% of the accessions in Pop2, along with the breeding lines or varieties, might indicate the early introduction of improved varieties in those regions, which might have latter been collected as landraces. Another possibility could be those accessions are landraces that might be used as breeding source for the development of advanced breeding lines or released varieties.

Although the STRUCTURE analysis showed two distinct subpopulations, there was a low level of pairwise differentiation between Pop1 and Pop2, which was justified by a PhiPT value of 0.07 (Table 3). AMOVA also indicated that variation between populations showed a significantly lower contribution to the total variation than within the population. As stated by Wright [53], gene flow (Nm) value is categorized into three categories high (Nm ≥ 1.0), medium (Nm = 0.25 0–0.99) and low (Nm = 0.0–0.249). In our study, Nm value > 1 was obtained, suggesting that high gene flow is a consequence of pollen flow or seed exchange by farmers, which could be one of the reasons for low differentiation between the two subpopulations. The Mantel test (Figure 8) also indicated the presence of gene flow since the genetic and geographic distance matrixes were not correlated. Similarly, Raman et al. [54] and Khedikar et al. [25] also found two distinct subpopulations from STRUCTURE analysis of 83 and 631 carinata populations, respectively, with a low genetic differentiation.

The genetic diversity indices, expected heterozygosity (*H_E_*), describe the proportion of heterozygous genotypes expected under Hardy–Weinberg equilibrium [55]. The larger the value of *H_E_*, the higher is variability or diversity of the populations. In the current study, *H_E_* value ranged from 0.1 to 0.5 with an average value of 0.314, indicating the presence of moderate diversity in carinata populations. In other similar studies, low to moderate diversity was reported for carinata and its related species *B. napus* [25,48,56,57]. With its long history of origin and cultivation, i.e., 4th to 5th century B.C [4], carinata is expected to have a high genetic diversity. However, the limited natural hybridization events and its geographical isolation, mainly in the East African highlands for many years, could contribute to its low to moderate genetic diversity [58]. Moreover, we found lower values of observed heterozygosity in comparison to expected heterozygosity, indicating its potential inbreeding among carinata genotypes, which in turn contribute to its low genetic diversity. The low value of observed heterozygosity was found to be prominent in Pop2 compared to Pop1 since the majority of Pop2 are breeding lines that could have higher inbreeding as compared to landraces or accessions, which are mainly found in Pop1.

PIC value is an important indicator of marker polymorphism among individuals of a population. The greater the PIC value, the better is the informativeness of the marker. It has enormous application for linkage analysis and other molecular breeding studies, such as genome-wide association (GWAS). Those markers with higher PIC value can be applied in GWAS to mark genes, alleles or haplotypes, which are linked to important quality traits. According to Botstein et al. [43], the SNP markers used in this study are moderately informative since the PIC value was found within the range of 0.25–0.50. This finding is to certain degree consistent with the previous studies by Khedikar et al. [25], and Thakur et al. [24] who reported an average PIC value of 0.24 and 0.29, respectively. We identified SNP markers that are more informative with a PIC value above the average and with a maximum PIC value of 0.38. The PIC value could significantly vary based on the type of markers used, the number of alleles per marker and the number and position of the markers in the chromosome [59]. Referring to the related species of *B. napus* and *B. juncea*, for example, the reported PIC value from diversity study using SSR marker was 0.43 and 0.39 by Wu et al. [60] and Singh et al. [61], respectively, while the PIC value using SNP markers were 0.22 for *B. napus* and 0.24 for *B. juncea* [48,49].

## 5. Conclusions

Single nucleotide polymorphisms (SNPs) are the most abundant markers and highly informative in investigating genetic diversity and genetic relationship among natural populations. Using high-density SNP markers, we were able to explore the genetic diversity and population structure of the 90 carinata genotypes. We employed 11,499 high-quality SNPs that cover 17 chromosomes for population structure and genetic diversity analysis from which two distinct populations (Pop1 and Pop2) were identified that showed higher variations within than between populations as well as low differentiation (PhiPT = 0.07) as revealed by AMOVA (*p* < 0.001). Low differentiation between subpopulations was likely due to the existence of gene flow as justified by Nm > 1 and the Mantel test with a low R-square value of 0.02, indicating weak geographical structure exists among the accessions. Moderate diversity was observed with an average expected heterozygosity of 0.31 and PIC of 0.26. Above all, the finding of this study enhances our understanding of the pattern of genetic diversity in carinata that would be useful for future breeding and conservation efforts of the crop.

## Figures and Tables

**Table 1:** The percentage of transition and transversion SNPs across the carinata genomes.

Genome	SNP Type	Model	No of SNP Loci	Frequency of SNPs (%)	Total SNP Number	SNPs (%)
B	Transition	A/G	3017	26.24	5990	52.09
		C/T	2973	25.85		
	Transversion	A/C	1187	10.32	2357	20.48
		G/T	1170	10.17		
C	Transition	A/G	1116	9.71	2195	19.09
		C/T	1079	9.38		
	Transversion	A/T	23	0.2	957	8.32
		A/C	458	3.98		
		G/T	457	3.98		
		G/C	19	0.17		

**Table 2:** Population distribution (Pop1 and Pop2) of carinata genotypes grouped based on the STRUCTURE analysis.

Source of Genotypes	Total Germplasms	Pop1		Pop2	
		Number	%	Number	%
Oromia	25	21	84.0	4	16.0
Amhara	25	13	52.0	12	48.0
SNNP*	10	6	60.0	4	40.0
SWEP*	5	4	80.0	1	20.0
BNG*	10	5	50.0	5	50.0
HARC*	15	0	6.7	14	93.3
Total	90	49	54.4	41	45.6

SNNP*: South Nation and Nationalities People, SWEP*: South West Ethiopian People, BNG*: Benishangul Gumuz, HARC*: Holetta Agricultural Research Center.

**Table 3:** Analysis of molecular variance (AMOVA), genetic differentiation (PhiPT) and gene flow (Nm) for 90 carinata genotypes.

**Source**	**DF**	**SS**	**MS**	**EV**	**%V**	**PhiPT**	**Nm**
Between populations	1	10,681.1	10,681.1	184.3	7%	0.07 **	6.65
Within populations	88	215,850.7	2452.8	2452.8	93%		
Total	89	226,531.8		2637.2	100%		

** *p*-value < 0.001. DF, degree of freedom. SS, sum of square. MS, mean of square. EV, error of variation. %V, percentage of variation.

**Table 4:** Genetic indices for estimation of genetic diversity in the analyzed carinata genotypes.

Genetic Indices	Carinata Genomes			Subpopulation	
	B	C	BC	Pop1	Pop2
Sample size	90	90	90	49	41
Number of SNPs	8347	3152	11,499	11,499	11,499
Genetic diversity/expected heterozygosity (*H_E_*)	0.315	0.309	0.314	0.317	0.299
Observed heterozygosity (*H_O_*)	0.179	0.168	0.176	0.257	0.079

**Table 5:** Average PIC value of 11,499 SNP markers across the B and C genomes of carinata.

B Genome			C Genome		
Chromosome	PIC	No. of SNPs	Chromosome	PIC	No. of SNPs
B1	0.27	731	C01	0.29	202
B2	0.26	1163	C02	0.22	296
B3	0.27	964	C03	0.27	580
B4	0.23	976	C04	0.18	652
B5	0.24	993	C05	0.30	236
B6	0.26	1022	C06	0.26	308
B7	0.26	1172	C07	0.30	420
B8	0.26	1326	C08	0.29	203
			C09	0.25	255
Mean	0.26		Mean	0.26	

## Data Availability

The data that support the findings of this study are available from the corresponding author upon reasonable request.

## Supplementary Materials

The following supporting information can be downloaded at: https://www.mdpi.com/article/10.3390/genes14091757/s1, Table S1: *B. carinata* genotypes used in the study; Table S2: Percentage of variation explained by the first three components and eigen values of the first five PCAs; Table S3: Genetic indices and polymorphism information content (PIC) of 11,499 SNP markers in *B. carinata* diversity analysis; Figure S1: Bar plot of STRUCTURE analysis for K = 3 (a), K = 4 (b) and K = 5 (c).
