# Supplementary figures and images for: Genetic Diversity and Population Structure in Ethiopian Mustard (Brassica carinata A. Braun) as Revealed by Single Nucleotide Polymorphism Markers
Source: Genes (Basel). 2023 Sep 3;14(9):1757. doi: 10.3390/genes14091757 (PMC10530317; doi:10.3390/genes14091757)

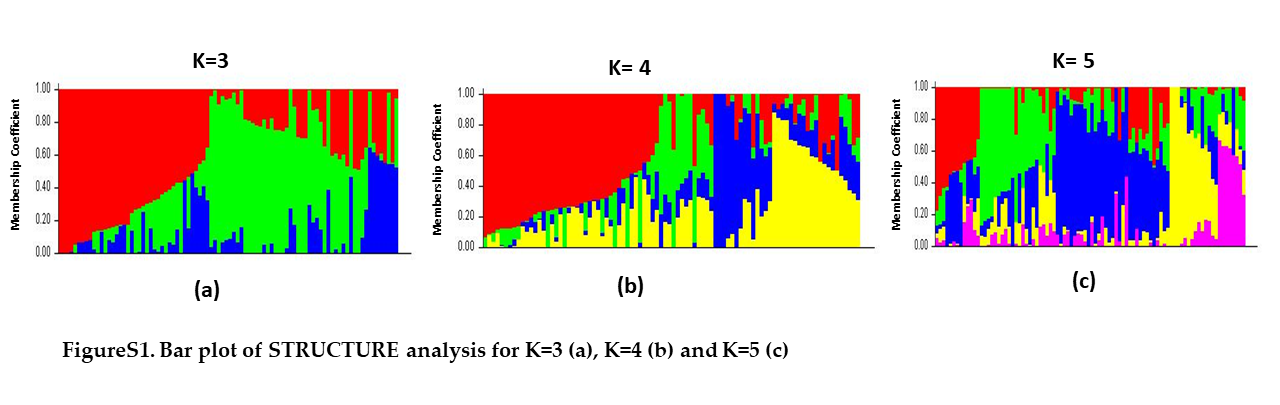

Supplement: Supplementary file 1 [file genes-14-01757-s001.zip › FigureS1. Bar plot of STRUCTURE analysis .tiff]
